# Validation of obesity based on self-reported data in Spanish women participants in breast cancer screening programmes

**DOI:** 10.1186/1471-2458-11-960

**Published:** 2011-12-30

**Authors:** Beatriz Isidoro, Virginia Lope, Carmen Pedraz-Pingarrón, Francisca Collado-García, Carmen Santamariña, Pilar Moreo, Carmen Vidal, María Soledad Laso, Milagros García-Lopez, Marina Pollán

**Affiliations:** 1 Cancer and Environmental Epidemiology Unit, National Centre for Epidemiology, Carlos III Institute of Health, Madrid, Spain; 2 Department of Preventive Medicine, Puerta de Hierro Majadahonda University Teaching Hospital, Madrid, Spain; 3 Consortium for Biomedical Research in Epidemiology & Public Health (CIBER en Epidemiología y Salud Pública - CIBERESP), Spain; 4 Castile-León Breast Cancer Screening Programme, General Directorate of Public Health (Gerencia Regional de Salud - SACyL), Castile-León, Spain; 5 Balearic Islands Breast Cancer Screening Programme, Health Promotion for Women and Children, General Directorate of Public Health & Participation, Regional Authority for Health & Consumer Affairs, Balearic Islands, Spain; 6 Galician Breast Cancer Screening Programme, Galician Regional Health Authority, Pamplona, Spain; 7 Aragon Breast Cancer Screening Programme, Aragon Health Service, Zaragoza, Spain; 8 Cancer Prevention and Control Unit, Catalonian Institute of Oncology (Institut Català d'Oncologia-ICO), Barcelona, Spain; 9 Valencian Breast Cancer Screening Programme, General Directorate of Public Health, Valencia, Spain; 10 Public Health Research Centre (Centro Superior de Investigación en Salud Pública -CSISP), Valencia, Spain; 11 Navarre Breast Cancer Screening Programme, Public Health Institute, Pamplona, Spain

## Abstract

**Background:**

Measurement of obesity using self-reported anthropometric data usually involves underestimation of weight and/or overestimation of height. The dual aim of this study was, first, to ascertain and assess the validity of new cut-off points, for both overweight and obesity, using self-reported Body Mass Index furnished by women participants in breast cancer screening programmes, and second, to estimate and validate a predictive model that allows recalculate individual BMI based on self-reported data.

**Methods:**

The study covered 2927 women enrolled at 7 breast cancer screening centres. At each centre, women were randomly selected in 2 samples, in a ratio of 2:1. The larger sample (n = 1951) was used to compare the values of measured and self-reported weight and height, to ascertain new overweight and obesity cut-off points with self-reported data, using ROC curves, and to estimate a predictive model of real BMI using a regression model. The second sample (n = 976) was used to validate the proposed cut-off points and the predictive model.

**Results:**

Whereas reported prevalence of obesity was 19.8%, measured prevalence was 28.2%. The sensitivity and specificity of this classification would be maximised if the new cut-off points were 24.30 kg/m2 for overweight and 28.39 kg/m2 for obesity. The probability of classifying women correctly in their real weight categories on the basis of these points was 82.5% in the validation sample. Sensitivity and specificity for determining obesity using the new cut-off point in the validation sample were 90.0% and 92.3% respectively. The predictive model for real BMI included the self-reported BMI, age and educational level (university studies vs lower levels of education). This model succeeded in correctly classifying 90.5% of women according to BMI categories, but its performance was similar to that obtained with the new cut-off points.

**Conclusions:**

Quantification of self-reported obesity entails a considerable underestimation of this problem, thereby questioning its validity. The new cut-off points established in this study and the predictive equation both allow for more accurate estimation of these prevalences.

## Background

Obesity, acknowledged as being as the epidemic of the 21st-century in the light of its sharp increase in industrialised countries [1], constitutes a growing health problem [2,3]. Obesity is associated with the development of a number of diseases, including breast cancer among post-menopausal women. Moreover, obesity ranks after smoking as the second leading risk factor for all-cause cancer [4]. In Spain, the prevalence of obesity among subjects aged over 18 years, obtained on the basis of self-reported data, stands at 16% [5] with evidence of a growing trend over the last two decades in men and women alike [6].

The collection of self-reported weight and height data by means of personal interviews, telephone calls or self-administered questionnaires, is frequently used in epidemiological studies to ascertain the prevalence of obesity, due mainly to their speed and low cost. Nevertheless, many authors have shown that self-reported weight and height values do not allow for a correct estimate of the prevalence of obesity, which leads to an underestimation of weight and/or overestimation of height, and, by extension, to an underestimation of Body Mass Index (BMI) [7-10]. In Spain, breast cancer screening programmes collect data on the principal risk factors for this tumour, including age, family history, main reproductive variables and, in many cases, self-reported weight and height.

There are studies in the literature which have performed corrections of self-reported BMI by developing equations or predictive models based on subjects' individual characteristics [8,11]. Knowledge of the factors that determine the underestimation of weight and overestimation of height, such as educational level, socioeconomic status or age are useful to correct understimated values [12], providing greater accuracy in estimating the prevalence of obesity and overweight.

Accordingly, the aim of this study was to assess the validity of self-reported anthropometric data furnished by women participants in breast cancer early detection programmes in Spain, propose new cut-off points for defining overweight and obesity categories and build up a predictive model to estimate real BMI from self-reported BMI. Finally, we validated the new cut-off points and the predictive model in an independent sample.

## Methods

### Sample

This study used the women enrolled in the DDM-Spain (Determinants of Mammographic Density in Spain - *Determinantes de la Densidad Mamográfica en España*) study, the goal of which was to investigate the prevalence of high mammographic density and their determinants in women participants in breast cancer early detection programmes in Spain [13]. Briefly, the study covered 3,574 women aged 45 to 68 years, at 7 screening centres in Aragon, the Balearic Isles, Castile-León, Catalonia, Galicia, Navarre and Valencia, with a minimum of 500 women per centre.

### Measure

Data were collected using a structured epidemiological questionnaire administered by a trained interviewer at the respective screening centres. Among other matters, participants were asked, "Could you tell us approximately how much you weigh?", and, "Could you tell us approximately how tall you are?". On completion of the survey, weight (in kg) and height (in cm) were measured twice by the interviewers, in accordance with standardised protocols and using identical types of scales and stadiometers at all centres. Where there was a wide divergence between the first two measurements, a 3^rd ^measurement was taken.

Standing height was measured using a stadiometer KaWe PERSON-CHECK^® ^(maximum height 200 cm; precision 0.5 cm). Weight was measured using a digital tilt Seca SA841^® ^(maximum weight 140 kg; precision 0.1 kg). Participants were requested to remove their shoes and any other heavy items of clothing and/or accessories that might constitute additional weight (coins, keys, etc.). BMI was calculated in accordance with the formula, "weight (kg)/height^2 ^(m)", for both real and self-reported measures. Overweight status was defined as BMI of 25-29.99 kg/m^2^, and obesity status as BMI ≥ 30 kg/m^2^, in line with the classification proposed by the World Health Organisation [14]. Women without any information on self-reported (643) or measured BMI (2) were excluded from the study.

The total sample (2927) was randomly divided into two groups: 2/3 of the population (1951 women) was allocated to sample I, i.e., the sample to be used for estimating the new cut-off points and the predictive equation. The remaining 1/3 (976 women) was allocated to sample II, reserved to validate the new cut-offs and the predictive model. Seeing as the women came from 7 different geographical settings, the process of random allocation in the established 2:1 ratio to samples I and II was performed separately at each.

Women who agreed to take part in the study signed an informed consent document, giving permission for their data to be included in databases for subsequent processing and analysis. At every phase of the study, respect was had for the basic ethical principles laid down by the prevailing Personal Data Protection Act (*Ley Orgánica de Protección de Datos de Carácter Personal*) [15]. The study was evaluated and approved by the ethics committee of Carlos III Institute of Health. The principles of the Declaration of Helsinki were respected.

### Statistics

The socio-demographic characteristics of the women in samples I and II were compared using Pearson's chi-squared test (for proportions) and Student's *t *test (for means). Sample I was used to assess the discrepancy between real and self-reported measures using the Student's *t *test for paired data. To evaluate graphically the agreement between self-reported and real BMI the method of Bland and Altman was used [16]. Factors associated with the difference between measured anthropometric data and self-reported data were identified using regression models taking the difference between real and self-reported BMI as the explanatory variable. Sample I was also used to propose new cut-off points for defining overweight and obesity categories based on self-reported data, using ROC curves.

We tested the validity of the new cut-off points in the validation sample, by estimating the percentage of correctly classified women and using a test for comparison of proportions to compare this percentage to that estimated on the basis of the traditional cut-off points. The concordance between weight categories according to measured and self-reported BMI under the new cut-off points was calculated by the weighted Kappa Index, using quadratic weights, which improved the degree of concordance established by the linear Kappa Index for ordinal variables, since it takes the distance between disagreements into account. The level of concordance was established using the Altman classification [17], which deems any value above 0.60 as indicative of high concordance.

In a second step, Sample I was used to generate a predictive equation to estimate real BMI in a linear regression model including self-reported BMI and those socio-demographic characteristics that influenced the differences observed between self-reported and real BMI. Women in the validation sample were then classified in the three categories, namely normal weight, overweight and obesity, taking into account their predicted BMI values. The performance of the model was assessed considering, as before, the percentage of women correctly classified, the weighted Kappa Index and ROC curves for obesity and overweight.

Statistical significance was set at a *p*-value < 0.05. All statistical procedures were performed using the Stata statistical software package version 10.0.

## Results

The overall study participation acceptance rate was 74.5%, with the lowest rate being recorded at the centre in Corunna (65%) and the highest in Zaragoza (84%). However, 18% of the women enrolled (643) could not be included in the study due to the fact that they failed to provide information on their height (81.2%), their weight (11.4%) or both (7.5%). Prevalence of obesity was significantly higher in these women than it was among those who were included in the study (35.8% versus 28.1%; *p *< 0.001).

The socio-demographic characteristics of the women in the study are shown in Table 1, with no significant differences being found between the study and validation samples.

**Table 1:** Socio-demographic characteristics of the women in samples I (study sample) and II (validation sample)

	Sample I	Sample II	*p*-value
		
Variables	N = 1951	N = 976	
		
	n (%)	n (%)	
**Age (in years)**			
45-49	327 (16.8)	135 (13.8)	
50-54	561 (28.8)	287 (29.4)	
55-59	537 (27.5)	287 (29.4)	
≥ 60	526 (27.0)	267 (27.4)	0.2135
**Self-reported BMI, mean (SD)**	26.6 (4.5)	26.5 (4.6)	0.5737
Self-reported weight	67.1 (11.8)	66.9 (11.8)	0.6656
Self-reported height	158.8 (6.0)	158.9 (6.1)	0.6725
**Measured BMI, mean (SD)**	27.8 (4.9)	27.8 (4.9)	1
Self-reported weight	68.5 (12.2)	68.4 (12.2)	0.8344
Self-reported height	156.9 (5.8)	157.0 (6.0)	0.6638
**Overweight**			
Based on self-reported BMI	772 (39.6)	342 (35.0)	0.0194
Based on measured BMI	831 (42.6)	412 (42.2)	0.8755
**Obesity**			
Based on self-reported BMI	387 (19.8)	206 (21.1)	0.4487
Based on measured BMI	550 (28.2)	270 (27.7)	0.7983
**Screening centre**			
Corunna (*La Coruña *)	309 (15.8)	168 (17.2)	
Barcelona	255 (13.1)	129 (13.2)	
Burgos	268 (13.7)	116 (11.9)	
Palma de Mallorca	336 (17.2)	174 (17.8)	
Pamplona	237 (12.2)	114 (11.7)	
Zaragoza	239 (12.3)	120 (12.3)	
Valencia	307 (15.7)	155 (15.9)	0.8418
**Town size**			
< 200,000	867 (44.4)	430 (44.1)	
200,000-300,000	517 (26.5)	272 (27.9)	
> 300,000	567 (29.1)	274 (28.1)	0.7064
**Marital status**			
Single	111 (5.7)	63 (6.5)	
Married/stable couple	1541 (79.9)	779 (79.8)	
Separated/divorced	167 (8.6)	75 (7.7)	
Widow	132 (6.8)	58 (5.9)	0.5766
**Menopause**			
Pre-menopausal	436 (22.4)	204 (20.9)	
Post-menopausal	1515 (77.7)	772 (79.1)	0.3982
**Educational level**			
Up to junior school-leaving certificate	1347 (69.1)	671 (68.8)	
Senior school-leaving certificate	198 (10.2)	93 (9.5)	
Vocational training	185 (9.5)	96 (9.9)	
University	219 (11.2)	115 (11.8)	0.9141
**Socio-economic level**			
Very low/low social class	469 (24.0)	223 (22.9)	
Middle social class	1383 (70.9)	689 (70.6)	
High/very high social class	92 (4.7)	60 (6.2)	0.2268

### Sample I: establishment of new cut-off points y estimation of predictive model

On average, measured weights were significantly greater than self-reported weights (Table 1). The oppposite is true regarding height, for which real measurements were significantly lower than self-reported ones. The mean difference between real and self-reported measurements was also statistically significant for both weight (1.41, SD 2.75, *p *< 0.001) and height (-1.92, SD 2.68, *p *< 0.001). Thirty two percent of women underestimated their weight two or more kilograms, and a 5% overestimated their height two or more cm. Consequently, self-reported BMI was substantially lower than BMI computed on the basis of measured data (self-reported BMI mean: 26.59 kg/m^2^, SD: 4.49, vs. measured BMI mean: 27.83 kg/m^2^, SD: 4.86; *p *< 0.001). In total, 52.3% of women underestimated their BMI one or more points.

**Figure 1:**
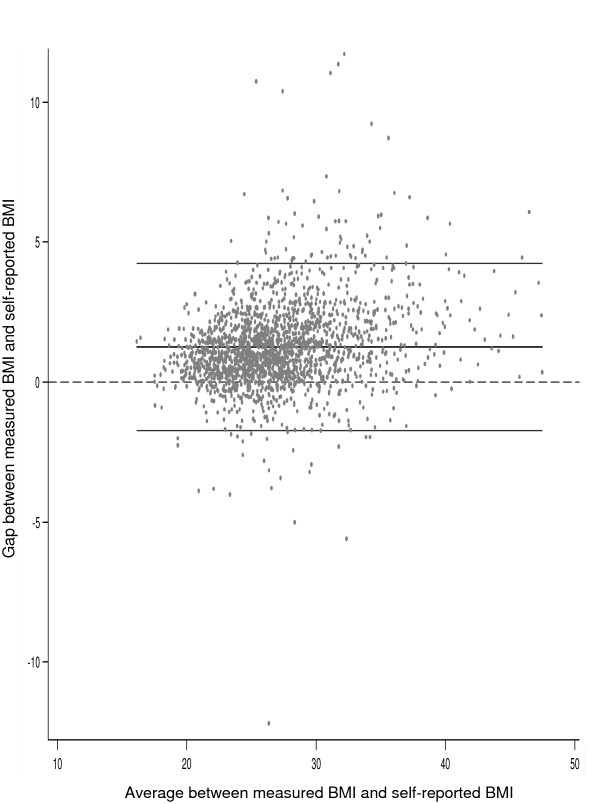
**Gap between measured and self-reported BMI compared to the average between measured and self-reported BMI**. Bland and Altman method.

Figure 1 shows the correlation between self-reported and measured BMI according to the Bland and Altman method. (Figure 1). The average difference between measured and self-reported BMI among women with normal weight was 0.61 (95% CI 0.52-0.71), and increased from 1.17 among overweight (95% CI 1.09-1.26) to 2.03 among obese women (95% CI 1.88-2.19). Self-reported data thus underestimated the prevalence of obesity (19.8% vs. 28.2%, *p *< 0.001) and, to a lesser extent, that of overweight (39.6% vs. 42.6%).

The sensitivity and specificity of self-reported BMI for the obesity classification threshold of 30 kg/m^2 ^was 68.18 (95% CI: 64.20-72.17) and 99.14 (95% CI 98.63-99.66), respectively. ROC curve analysis furnished an optimal obesity cut-off point for self-reported BMI of 28.39 kg/m^2^, thereby increasing sensitivity (88.36; 95% CI 85.59-91.13). Specificity was 94.65 (95% CI 93.43-95.86). The area under the ROC curve improved from 0.84 (95% CI 0.82-0.86) to 0.92 (95% CI 0.91-0.93), with this difference proving statistically significant (*p *< 0.001).

In the case of overweight, the original cut-off point of 25 kg/m^2 ^displayed a sensitivity of 82.6% (95% CI: 80.9%-84.3%), which improved substantially with the new overweight threshold established at 24.30 kg/m^2^, (sensitivity 91.09, 95% CI 89.55-92.63; specificity 90.18, 95% CI 87.64-92.71). Nevertheless, the area under the ROC curve increased only discreetly, going from 0.90 (95% CI 0.88-0.91) to 0.91 (95% CI 0.89-0.92), with this difference not proving statistically significant (*p *= 0.161).

Factors associated with the difference between measured anthropometric data and self-reported data are shown in Table 2. The underestimation of BMI was more pronounced in older women and in women with higher BMI (*p *= 0.013 and *p *< 0.001, respectively). After adjusting by age and measured BMI, educational level was the only sociodemographic variable that proved to contribute to the gap between self-reported and measured values (*p *= 0.014). As it can be seen in Table 2, differences between measured and self-reported BMI were similar among women in the first three categories of education, but those with university studies underestimated their BMI considerably less. Finally, differences between self-reported and measured BMI varied among the screening centres.

**Table 2:** Factors associated with the difference between measured and self-reported anthropometric data

Variables	Mean Difference*	95%IC	*p*-value**
**Measured IMC**			
Underweight and Normal weight	0.59	0.50-0.69	
Overweight	1.17	1.09-1.26	
Obesity	2.03	1.88-2.19	< 0.001
**Age**			
45-49	0.98	0.83-1.12	
50-54	1.02	0.89-1.16	
55-59	1.32	1.19-1.45	
≥ 60	1.48	1.36-1.59	0.013
**Town size**			
> 200,000	1.30	1.20-1.38	
200,000-10,000	1.17	1.06-1.28	
< 10,000	1.28	1.01-1.54	0.648
**Marital status**			
Single	1.28	1.02-1.53	
Married/stable couple	1.24	1.16-1.32	
Separated/divorced	1.19	0.97-1.41	
Widow	1.38	1.12-1.64	0.283
**Menopause**			
Pre-menopausal	0.97	0.83-1.11	
Post-menopausal	1.32	1.25-1.40	0.530
**Educational level**			
Up to junior school-leaving certificate	1.29	1.20-1.37	
Senior school-leaving certificate	1.34	1.11-1.58	
Vocational training	1.24	1.02-1.46	
University	0.93	0.77-1.10	0.014
**Socio-economic level**			
Very low/low social class	1.26	1.11-1.41	
Middle social class	1.25	1.17-1.33	
High/very high social class	1.11	0.89-1.34	0.188
**Screening centre**			
Corunna (*La Coruña *)	1.61	1.40-1.82	
Barcelona	1.23	1.06-1.40	
Burgos	0.72	0.54-0.90	
Palma de Mallorca	1.62	1.48-1.76	
Pamplona	1.59	1.41-1.77	
Zaragoza	0.58	0.42-0.73	
Valencia	1.20	1.03-1.37	< 0.001

* Measured BMI - Self-reported BMI

** Data are adjusted by age and measured BMI

A regression model was then fitted to estimate BMI from self-reported data, age and level of education. This last factor was dichotomized considering women without and with university studies. Continuous variables were centred and interactions between these explanatory variables were tested, being none of them statistically significant. Self-reported BMI, age and university studies explained 90.5% of the variability observed in the measured BMI. The final model was as follows: BMI = 26.90 + 1.02 (self-reported BMI - 25.64) + 0.04 (age - 55.91) - 0.23 (university Studies = yes).

### Sample II: validation results

The validation sample comprised 976 women. The prevalences of obesity and overweight estimated on the basis of measured data were 27.7% and 42.2%, as compared to 21.1% and 35.0% respectively when estimated on the basis of self-reported data.

**Figure 2:**
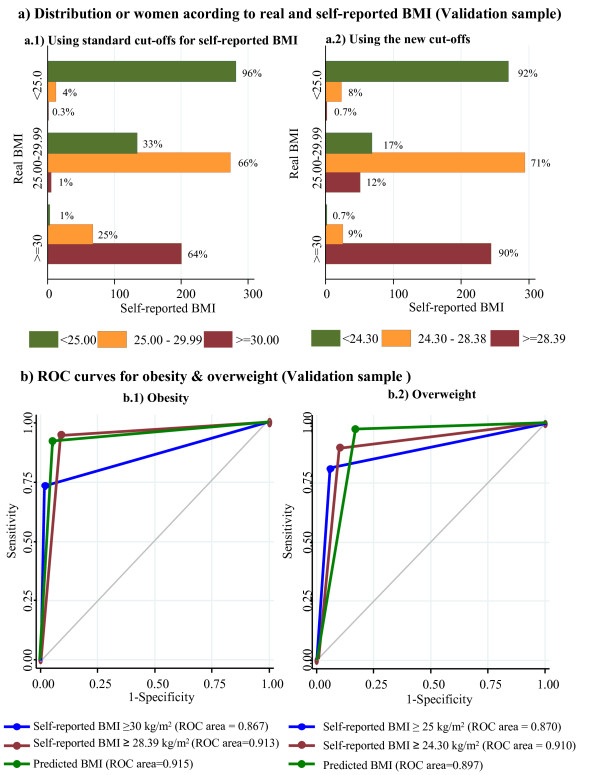
**a) Classification of women in the validation sample using self-reported data and traditional cut-off points (left), and new cut-off points (right)**. **b**) ROC curves for obesity (left) and overweight (right) in the validation sample using self-reported data with traditional cut-off points, those estimated in our study and predicted BMI.

Figure 2a depicts the distribution by real weight categories according to self-reported BMI, using the traditional (Figure 2a.1) and new cut-off points (Figure 2a.2) and the first two columns in Table 3 compare the performance of self reported BMI with the classification using new cut-offs points. Figure 2a shows that, whereas using the new cut-off points to ascertain the presence of obesity displayed a sensitivity of 90.0% (95% CI 85.8-93.3) and a specificity of 92.3% (95% CI 90.1-95.0), using the traditional cut-off points yielded a sensitivity of 64.0% (95% CI 61.0-97.1) and a specificity of 96.0% (95% CI 94.7-97.3). The proportion of overweight women correctly classified by reference to self-reported BMI also increased when the new cut-off points were used (71.0%, 95% CI 68.1-73.9 vs. 66.0%, 95% CI 63.0-69.0). A total of 82.5% of women were classified correctly in their weight categories, 6.3% more than when using the traditional cut-off points (Table 3), with this gain being statistically significant (p-value = 0.004). In the case of obesity, the positive predictive value was 82.09 (95% CI 77.56-86.63) and the negative predictive value was 96.03 (95% CI 94.49-97.57); in the case of overweight, these values were 85.92 (95% CI 82.09-89.76) and 81.26 (95% CI 78.15-84.37) respectively. A high degree of concordance was observed between the classification using anthropometric measures and self-reported data with the new cut-off points (weighted kappa of 0.85; 95% CI: 0.82-0.87). Finally, there was a statistically significant gain in the discriminative power of the new cut-offs for obesity and for overweight proved by the observed differences in ROC areas (Table 3; *p *< 0.001).

**Table 3:** Performance of self-reported BMI, self-reported BMI using the new cut-offs and the predictive regression model in the validation sample

	Self reported BMI	Using the new cut-offs	Regression model
% correctly classified (95% CI)	76.2% (73.4-78.9)	82.5% (79.9-84.8)	84.6% (82.2-86.8)
Weighted kappa (95% CI)	0.79 (0.77-0.82)	0.85 (0.82-0.87)	0.86 (0.84-0.88)
ROC Area Obesity (95% CI)	0.87 (0.84-0.89)	0.91 (0.89-0.93)	0.92 (0.89-0.94)
ROC Area Overweight (95% CI)	0.87 (0.85-0.89)	0.91 (0.89-0.93)	0.90 (0.88-0.92)

Regarding the validation of the predictive model, the mean of predicted BMI values was 27.77 (SD 4.62) very similar to the mean of the measured BMI in this group of women (mean = 27.79; SD = 4.92). The performance of this model is presented in the last column of Table 3. Using the predicted values yielded by the regression model, the percentage of women correctly classified into their corresponding weight categories was 84.6%, this percentage is not significantly different to that obtained using the new cut-off points (Table 3). The Kappa index presented a non significant increase of a 1% (Weighted kappa: 0.86; 95% CI: 0.84 to 0.88). The area under the ROC curve also were very similar to those obtained with the proposed cut-offs (Figure 2b).

## Discussion

Our results confirm the underestimation of prevalence of overweight and obesity when self-report measures are used in Spanish women attending breast cancer screening programs. Validation of self-reported data in this population had not been previously reported. Prevalence of obesity calculated on the basis of self-reported values was 19.8%, which differed substantially from the prevalence of real obesity, which stood at 28.2%. In the latest European Health Survey (*Encuesta Europea de Salud *2009), prevalence of self-reported obesity among Spanish women aged 45-64 years was 17.3% [5]. Our sample of women thus displayed high obesity indices.

Techniques of collecting self-reported data have also been analysed by some authors. As compared to personal interviews, the telephone interview has been frequently associated with larger underestimates of prevalence of obesity [18]. The impossibility of an interviewer verifying the information furnished by telephone might account for this phenomenon. Nevertheless, Galán et al. [19] highlighted the fact that the results of telephone and "face-to-face" interviews were very similar. At all events, the heterogeneity observed reveals the importance of validating self-reported information, so that the reliability of the data is ensured.

Many studies have observed substantial differences between measured and self-reported values, though the magnitude of the discrepancy is variable [7,8,10,20,21]. In our study, more than 50% of women underestimated their BMI one or more points, with these results once again being higher than those reported by other authors [7,8]. Among the factors that explain this difference are measured weight and height, since persons with elevated weight and short stature tend to underestimate their BMI, probably due to personal desire of thinner and more slender body image, especially in women [9,22]. In line with other studies, we observed a tendency to underestimate BMI with an increase in women's age [10,23] and a more accurate self-reported BMI in women with university studies [6,24]. Other factors, such as size of municipality, marital status or socio-economic level, did not significantly influence the differences observed between measured and self-reported BMI. However, there was substantial heterogeneity between screening centres regarding the amount of underreporting, something that may be related with other social or cultural characteristics not considered in our study [9.12].

Although many studies confirm the underestimation inherent in self-reported data, few provide the possibility of using new cut-off points when it comes to using self-report measures. In our study, despite the fact that the specificity of self-reported BMI was good (99%), there was clearly insufficient capacity to ascertain obesity status (sensitivity of 68.2%). Similar findings about the new cut-off point have been reported by other studies [10,25]. Dauphinot et al. [7] propose a new obesity cut-off point of 29.2 kg/m^2^, higher than that obtained by us, though it is important to highlight the difference in the prevalence of obese women that separates the two samples. Another fact established by our study is the high degree of concordance between weight categories achieved when using measured BMI and self-reported BMI with the new cut-off points. It is greater than that found in similar analyses [20,25]. This degree of agreement was also confirmed in our study with the validation sample.

Other studies have previously indicated that deviations in self-reported data depend on demographic, cultural, social and health characteristics of a population at any given time [9.12]. Therefore, we tried to develop a predictive model from self-reported values, with an account of some of these factors, such as age and educational degree. Bolton-Smith et al. [8] proposed a model which is very consistent with that presented here, also including age and self-reported weight and height. Nevertheless, in our case our model did not perform better than the simple adoption of the new cut-offs.

Our study sought to quantify the degree to which obesity was underreported by women participants in breast cancer screening programmes in Spain. Obesity is a risk factor for breast cancer in postmenopausal women, so that many of the screening programmes record weight and height reported by women outpatients undergoing screening mammography. The newly proposed cut-off points could be useful in the context of such programmes. It is important to bear in mind, however, that this study included women from only 7 screening centres, which, albeit situated in different geographical settings, might not adequately represent the variability of our target population. Our study's principal limitation stems from the selection of the study sample. On the one hand, subjects had to agree to participate, so that self-selection could threaten the generalisability of the results; and on the other, the prevalence of obesity among the women who were not included in the study due to failure to furnish anthropometric data, was significantly higher than that among the women who were included (35.8 vs 28.1%; *p *< 0.001), which suggests that women with larger BMI were underrepresented in our study. Older women, women with higher measured BMI, women with lower education and women living in rural areas were less able to provide the necessary information to compute their BMI. Interestingly, in most cases (81.2% of women without self-reported BMI) answered the question regarding their weight but did not know their height, 73 women (11.4%) reported their height but not their weight and only 7% of them did not provided any information. Taking these data into account it seems that younger women, urban women and women with a higher educative level are more able to report their height and probably also know more about BMI as an overall measure of obesity.

The applicability of the new cut-off points and the proposed predictive model to other populations must be approached with care. Our study included a variety of screening centres across the country, providing an overall picture regarding the underestimation of BMI and the factors associated with it. However, differences between centers in our study were not fully explained by the heterogeneous distribution of age and other explanatory variables among them. To what extent these differences are related to unmeasured socio-demographic or cultural characteristics or reflect differences in the way the study was carried out is impossible to know. Given the geographical dispersion among centers, a different interviewer was used in each of them. These women were trained by the study coordinator who also periodically supervised their work. However, in spite of the training, it is possible that part of the heterogeneity among screening centres could be caused by differences in the way interviewers gathered information or even weighted and measured the participants. In summary, the validity of our results should be explored before being applied to other centres.

Despite these limitations and having regard to the degree of underreporting observed, the newly proposed cut-off points could nevertheless prove useful for ascertaining overweight and obesity in women attending screening programmes. Obesity is a known risk factor for many chronic diseases [26-28], including breast cancer among post-menopausal women [29]. The high prevalence of obesity detected among the women in our study makes them a risk group to be borne in mind in prevention programmes. Visit to the screening centre could well be a useful time to raise women's awareness as to overweight-related problems and motivate them to achieve a healthier BMI.

## Conclusions

The results of our study confirm the underestimation of obesity in Spanish women attending breast cancer screening when self-reported data are used, and suggest the possibility of using new cut-off points to assess the presence of overweight and obesity on the basis of this type of information. Enhancing the sensitivity of the measure would serve to provide more realistic information on the magnitude of the problem of obesity. In view of the high prevalence of obesity detected by us, peri- and post-menopausal women who attend breast cancer screening constitute a risk group.

## Competing interests

The authors declare that they have no competing interests.

## Authors' contributions

BI and VL: Study design, analysis and interpretation of results, and draft the manuscript. CPP, FCG, CS, PM, CV, MSL and MGL: Study design, data-collection and critical review of the manuscript (senior screening-programme research). MP: Study design, design of research, analysis and interpretation of results, contribution to manuscript writing and review it for important content (senior researcher of the team). All the authors read and approved the final manuscript.

## Query

Q1: City "Pamplona" has been inserted for "affiliations 3 and 6". Please check and correct if necessary.

## Pre-publication history

The pre-publication history for this paper can be accessed here:

http://www.biomedcentral.com/1471-2458/11/960/prepub
